# Inhibition of hypoxia inducible factor 1 and topoisomerase with acriflavine sensitizes perihilar cholangiocarcinomas to photodynamic therapy

**DOI:** 10.18632/oncotarget.6490

**Published:** 2015-11-27

**Authors:** Ruud Weijer, Mans Broekgaarden, Massis Krekorian, Lindy K. Alles, Albert C. van Wijk, Claire Mackaaij, Joanne Verheij, Allard C. van der Wal, Thomas M. van Gulik, Gert Storm, Michal Heger

**Affiliations:** ^1^ Department of Experimental Surgery, Academic Medical Center, University of Amsterdam, 1105 AZ Amsterdam, The Netherlands; ^2^ Department of Controlled Drug Delivery, MIRA Institute for Biomedical Technology and Technical Medicine, University of Twente, 7500 AE Enschede, The Netherlands; ^3^ Department of Pathology, Academic Medical Center, University of Amsterdam, 1105 AZ Amsterdam, The Netherlands; ^4^ Department of Pharmaceutics, Utrecht Institute for Pharmaceutical Sciences, University of Utrecht, 3584 CG Utrecht, The Netherlands

**Keywords:** cancer therapy, drug delivery system, extrahepatic cholangiocarcinoma, hypoxia, tumor targeting

## Abstract

**Background:** Photodynamic therapy (PDT) induces tumor cell death by oxidative stress and hypoxia but also survival signaling through activation of hypoxia-inducible factor 1 (HIF-1). Since perihilar cholangiocarcinomas are relatively recalcitrant to PDT, the aims were to (1) determine the expression levels of HIF-1-associated proteins in human perihilar cholangiocarcinomas, (2) investigate the role of HIF-1 in PDT-treated human perihilar cholangiocarcinoma cells, and (3) determine whether HIF-1 inhibition reduces survival signaling and enhances PDT efficacy.

**Results:** Increased expression of VEGF, CD105, CD31/Ki-67, and GLUT-1 was confirmed in human perihilar cholangiocarcinomas. PDT with liposome-delivered zinc phthalocyanine caused HIF-1α stabilization in SK-ChA-1 cells and increased transcription of HIF-1α downstream genes. Acriflavine was taken up by SK-ChA-1 cells and translocated to the nucleus under hypoxic conditions. Importantly, pretreatment of SK-ChA-1 cells with acriflavine enhanced PDT efficacy via inhibition of HIF-1 and topoisomerases I and II.

**Methods:** The expression of VEGF, CD105, CD31/Ki-67, and GLUT-1 was determined by immunohistochemistry in human perihilar cholangiocarcinomas. In addition, the response of human perihilar cholangiocarcinoma (SK-ChA-1) cells to PDT with liposome-delivered zinc phthalocyanine was investigated under both normoxic and hypoxic conditions. Acriflavine, a HIF-1α/HIF-1β dimerization inhibitor and a potential dual topoisomerase I/II inhibitor, was evaluated for its adjuvant effect on PDT efficacy.

**Conclusions:** HIF-1, which is activated in human hilar cholangiocarcinomas, contributes to tumor cell survival following PDT *in vitro*. Combining PDT with acriflavine pretreatment improves PDT efficacy in cultured cells and therefore warrants further preclinical validation for therapy-recalcitrant perihilar cholangiocarcinomas.

## INTRODUCTION

Photodynamic therapy (PDT) is a non-to-minimally invasive treatment modality for a variety of solid cancers. This therapy is based on the accumulation of a light-sensitive drug (photosensitizer) in the tumor following systemic administration. Next, the photosensitizer-replete tumor is locally irradiated with (laser) light, resulting in the activation of the photosensitizer and subsequent production of reactive oxygen species (ROS) via type I (superoxide) and/or type II (singlet oxygen) photochemical reactions. Consequently, PDT locally induces a state of hyperoxidative stress, culminating in tumor cell death, destruction of the microvasculature that causes tumor hypoxia and hyponutrition, and an anti-tumor immune response [1, 2].

PDT is effective in the curative treatment of (pre-) malignant skin lesions (actinic keratosis, basal/squamous cell carcinoma) [3], but is also employed as (last-line) treatment of head and neck cancer [4], early central stage lung tumors [5], esophageal cancer [6], nasopharyngeal carcinomas [7], bladder cancer [8], and non-resectable perihilar cholangiocarcinomas [9]. Although PDT yields complete response rates of 50–90% in the majority of the abovementioned cancers, nasopharyngeal-, urothelial-, and perihilar cholangiocarcinomas are relatively refractory to PDT. This may be in part due to hypoxia-mediated survival signaling that is triggered by the stabilization of hypoxia inducible factor 1 (HIF-1) following PDT [10–12]. In nasopharyngeal and superficial urothelial carcinomas, the overexpression of HIF-1α has been associated with poor overall survival [13, 14]. HIF-1 expression levels in perihilar cholangiocarcinomas are currently elusive but may account for the recalcitrance of these tumors to therapy [15].

HIF-1 is a transcription factor composed of HIF-1α and HIF-1β subunits. During normoxia, prolyl-hydroxylases (PHD) and factor inhibiting HIF (FIH) mediate the hydroxylation of Pro402, Pro564, and/or Asn803 of HIF-1α [16]. In turn, Von Hippel-Lindau tumor suppressor protein (VHL) binds to hydroxylated HIF-1α, resulting in complexation with E3 ubiquitin ligase and subsequent proteasomal degradation of HIF-1α [17, 18]. In contrast, hypoxia inhibits the activity of both PHDs and FIH, leading to HIF-1α stabilization and nuclear translocation [19]. After translocation to the nucleus, HIF-1α dimerizes with HIF-1β and mediates the transcription of various genes [20] that are involved in glycolysis, angiogenesis, survival, and apoptosis [21–23]. Alternatively, HIF-1 may be activated through ROS, which also deter the activity of PHDs and FIH, leading to the stabilization and nuclear translocation of HIF-1α [24, 25].

HIF-1 is constitutively active in most tumors since the tumor growth rate exceeds the rate of neoangiogenesis [21, 23]. Moreover, HIF-1 is responsible for resistance to chemotherapy and radiotherapy [26, 27]. PDT increases HIF-1 activity in mouse mammary carcinoma (EMT-6) cells [28] and human bladder cancer (UROtsa, RT112, and J84 but not RT4) cells [29] as well as in murine Kaposi's sarcoma- [30], BA mouse mammary carcinoma- [31, 32], and CNE2 nasopharyngeal carcinoma xenografts [33]. Inhibition of HIF-1 activity and corollary survival signaling may consequently improve the therapeutic efficacy of PDT.

This study therefore investigated the therapeutic potential of the HIF-1 dimerization inhibitor acriflavine (ACF) in an *in vitro* PDT setting for the treatment of human perihilar cholangiocarcinoma (SK-ChA-1) cells [34], *i.e.*, a cell line derived from a type of cancer that is recalcitrant to different types of treatment. The photosensitizer used in this study was zinc phthalocyanine (ZnPC), a second-generation photosensitizer that was encapsulated in cationic liposomes designed to target tumor cells and tumor endothelium [35, 36]. ACF was selected due to its selective inhibition of HIF-1 activation [37] and due to its clinical safety [38]. In a recent study, it was shown that ACF downregulates the HIF-1 target gene vascular endothelial growth factor (*VEGF*) and reduces the amount of tumor microvessels in murine breast carcinoma (4T1)-bearing mice [39]. Moreover, Wong *et al*. revealed that treatment of human breast carcinoma (MDA-MB-231 and MDA-MB-435)-xenografted mice with ACF inhibited HIF-1-mediated invasion and metastasis [40]. Besides HIF-1 inhibition, ACF was also investigated in the context of its dual topoisomerase I and II inhibitor activity, as discovered by Hassan *et al*. [41]. Topoisomerases are involved in the cleavage and resealing of DNA breaks during transcription and cell replication, and inhibition of these topoisomerases may lead to cell cycle arrest and apoptosis in dividing cells (reviewed in [42]).

The most important findings were that HIF-1 is activated by sublethal PDT in SK-ChA-1 cells. Immunostaining of patient-derived perihilar cholangiocarcinoma biopsies demonstrated extensive neovascularization in desmoplastic tissue and heterogeneous glucose transporter 1 (GLUT-1) overexpression, hinting towards the possible involvement of hypoxia- and HIF-1-mediated angiogenesis. *In vitro*, pretreatment of tumor cells with ACF improved PDT outcome and reduced the PDT-induced expression of *VEGF* and *PTGS2*. Lastly, incubation of SK-ChA-1 cells with ACF resulted in induction of S-phase cell cycle arrest, DNA damage, and apoptosis, altogether underscoring ACF's dual topoisomerase I/II inhibition potential and utility to act as a neoadjuvant chemotherapeutic in PDT.

## RESULTS

### Expression of hypoxia-related proteins in human perihilar cholangiocarcinoma

**Figure 1:**
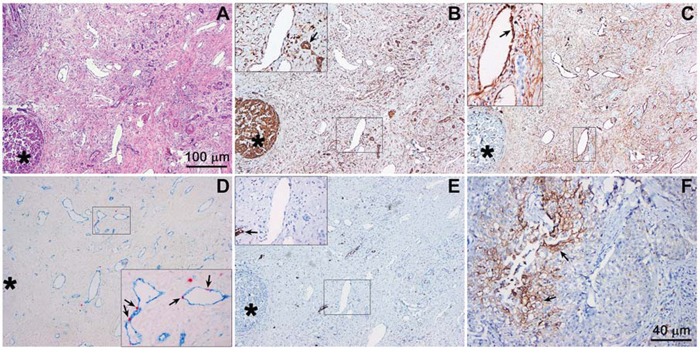
Hypoxia-related protein expression in an extrahepatic perihilar cholangiocarcinoma (Klatskin tumor) resection specimen Serial histological sections were used for protein profiling in the same region. **A.** Hematoxylin and eosin staining of a cholangiocarcinoma section containing tumor mass (intense purple staining, circular structure, bottom left, marked with an asterisk in panels A-E), tumor stroma, and native tissue (*e.g.*, pre-existent arterial structures). **B.** VEGF staining (brown), showing intense staining of the tumor mass and vascular endothelium (insert) as well as pre-existent biliary structures (insert, arrow). The insert corresponds to the demarcated region in the low-magnification image. **C.** CD105 staining (brown), showing no staining in the tumor mass and positive staining of the vascular endothelial cells in the tumor stroma (insert, arrow, indicates neovessel formation). **D.** Angiogenesis was further confirmed with CD31 (blue) and Ki-67 (red) double staining, showing that the blood vessels in the tumor stroma contain proliferating endothelial cells (insert, arrows). **E.** GLUT-1 staining (brown) was largely absent in the tumor mass and stroma, indicating that these regions were not affected by hypoxia. In some regions of the tumor, however, positive staining was observed (insert, arrow). **F.** Strong GLUT-1 staining was found in another region of the histological specimen. Magnification: 4× (A-E, scale bar = 100 μm) and 10× (F, scale bar = 40 μm).

Although the incidence of tumor hypoxia and the importance of HIF-1 expression in a large variety of tumors have been widely established, literature on this phenomenon in perihilar cholangiocarcinomas is scarce. Therefore, it was investigated whether hypoxia-related proteins (VEGF for angiogenic signaling, CD105 and CD31/Ki-67 for neovascularization, and GLUT-1 for glycolysis) were present in perihilar cholangiocarcinoma resection specimens. Of note, immunostaining for HIF-1α directly was not performed due to its high instability (protein half-life of 5–8 minutes) [43]. Representative differently stained serial images are presented in Figure 1.

The hematoxylin and eosin staining (Figure 1A) revealed that perihilar cholangiocarcinomas were characterized by clusters of tumor cells surrounded by relatively large areas of desmoplastic tissue (*i.e.*, stroma). The tumor mass stained positively for VEGF (as did liver tissue), whereas VEGF staining was less prominent in the tumor stroma (Figure 1B). Nevertheless, the tumor stroma was densely vascularized. The vasculature in the desmoplastic tissue was not of pre-existent nature, as the endothelium stained positively for CD105, a marker for angiogenic endothelium (Figure 1C), and Ki-67, a marker of proliferation (Figure 1D). Of note, the tumor mass was largely devoid of Ki-67-positive cells, indicating that the perihilar cholangiocarcinomas in our patient population were slowly proliferating tumors. GLUT-1 was largely absent in the tumor cell mass and stroma (Figure 1E), albeit several regions containing GLUT-1-expressing cell clusters were observed in other sections of the tumor (Figure 1F). Accordingly, these results provide compelling evidence for the presence of hypoxia and HIF-1 activation in perihilar cholangiocarcinomas, which likely drive angiogenesis and regional upregulation of glycolysis. Moreover, the perihilar cholangiocarcinomas are replete with vasculature that may serve as a conduit for the delivery of liposome-encapsulated photosensitizers.

### HIF-1 is activated after PDT

**Figure 2:**
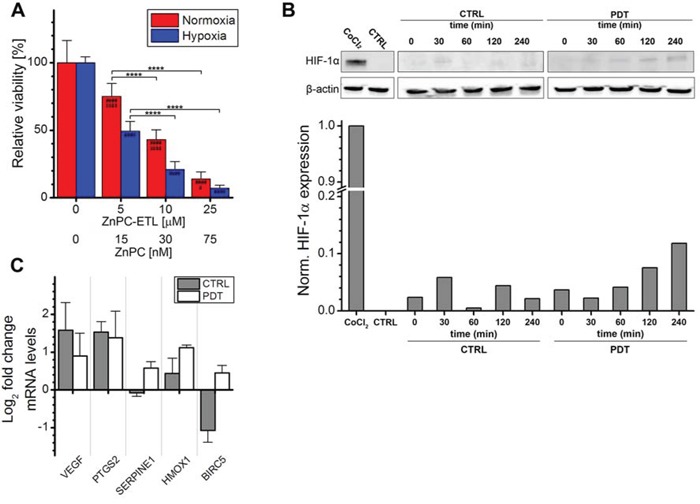
Analysis of HIF-1α activation after PDT **A.** SK-ChA-1 cells were incubated with increasing concentration of ZnPC-ETLs, treated with PDT, and maintained at normoxic (red bars) or hypoxic (blue bars) culture conditions. Cell viability was determined 24 hours post-PDT (*n* = 6 per group). **B.** SK-ChA-1 cells were treated with PDT (10 μM ZnPC-ETLs, final lipid concentration) or received a control (CTRL) treatment, after which the cells were placed in a hypoxic chamber up to 240 minutes (min) post-PDT. HIF-1α protein levels were determined using Western blotting. As a positive control, cells were incubated with 500 μM CoCl_2_ for 24 hours (top panel). Next, the HIF-1α protein bands and their corresponding β-actin protein bands were quantified using ImageJ software [74] and each HIF-1α value was divided by its corresponding β-actin value. All values were normalized to the positive control (CoCl_2_) (bottom panel). **C.** SK-ChA-1 cells were either left untreated (grey bars) or treated with PDT (white bars), and subsequently placed at hypoxic conditions for 4 hours. Thereafter, downstream targets of HIF-1 were analyzed with qRT-PCR (*n* = 3 per group). Readers are referred to the experimental section for the significance of the statistical symbols.

To establish whether HIF-1 was activated by PDT, the optimal PDT dose was first determined in perihilar cholangiocarcinoma (SK-ChA-1) cells. SK-ChA-1 cells were incubated with increasing concentrations of ZnPC-encapsulating cationic liposomes (ZnPC-ETLs) and subsequently treated with PDT (500 mW, 15 J/cm^2^). These liposomes have been shown to selectively accumulate in tumor endothelium [44], which is expected to translate to vascular shutdown and exacerbated tumor hypoxia following PDT [15]. Moreover, the ZnPC-ETLs are taken up by tumor cells, including SK-ChA-1 cells (manuscript in preparation). After PDT, the cells were either maintained under normoxic or hypoxic culture conditions (Figure 2A) to mimic the PDT-induced vascular shutdown [45, 46]. Cell viability was determined 24 hours after PDT using the WST-1 assay. Cells exhibited a ZnPC concentration-dependent decrease in cell viability following PDT, whereby the extent of cell death was exacerbated by hypoxia (Figure 2A). Since the IC_50_ concentration in normoxic and hypoxic cells were approximately 10 and 5 μM ZnPC-ETLs (final lipid concentration), respectively, these concentrations were used in the rest of the experiments.

Next, the stabilization of HIF-1α and induction of HIF-1α transcriptional targets were investigated following PDT. As shown in Figure 2B, normoxic SK-ChA-1 cells exhibited no notable HIF-1α expression. Stimulation of cells with cobalt chloride is commonly used to induce hypoxic signaling [47, 48] and was therefore used as positive control. Indeed, cobalt chloride caused extensive HIF-1α stabilization. Accordingly, SK-ChA-1 cells that were placed in a hypoxic chamber stabilized HIF-1α in a time-dependent manner, albeit less extensively than after cobalt chloride stimulation. HIF-1α stabilization was enhanced upon PDT.

The HIF-1α stabilization was associated with upregulated transcription of several HIF-1 target genes, including *VEGF* (angiogenesis), *PTGS2* (survival), and *HMOX1* (survival) (Figure 2C). SK-ChA-1 cells also upregulated *SERPINE1* (angiogenesis) and baculoviral inhibitor of apoptosis repeat-containing 5 (*BIRC5*, survival) after PDT. It was therefore concluded that HIF-1α is upregulated in SK-ChA-1 cells following PDT, albeit to a minor extent in comparison to the cobalt chloride treatment.

### ACF is translocated to the nucleus upon hypoxia and/or PDT

**Figure 3:**
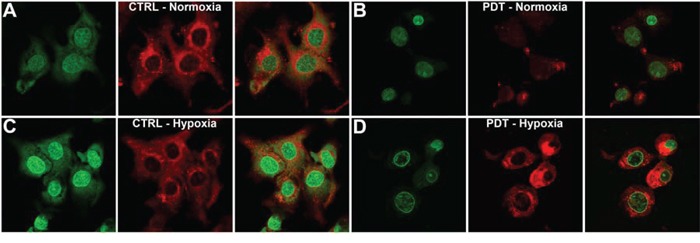
Intracellular ACF localization **A–D.** SK-ChA-1 cells were either left untreated or treated with PDT and subsequently incubated with ACF for 4 hours under normoxic (A, B) or hypoxic culture conditions (C, D). ACF localization was determined using confocal microscopy (ACF in green; Nile Red (membrane staining) in red).

Since PDT induced HIF-1 signaling in SK-ChA-1 cells, which may be responsible for the therapeutic recalcitrance *in vivo*, we investigated whether the HIF-1α/HIF-1β dimerization inhibitor ACF would enhance PDT efficacy. First, the intracellular localization of ACF was determined by confocal microscopy, whereby the intrinsic fluorescence of ACF (λ_ex_ = 453 nm and λ_em_ = 507 nm) in combination with (intra)cellular membrane staining (Figure 3). SK-ChA-1 cells were incubated with ACF during normoxia, hypoxia, and/or after PDT to study the cytosolic-to-nuclear translocation of ACF during these processes.

As shown in Figure 3A, ACF was localized in both the nucleus and cytosol under normoxic conditions. PDT treatment was accompanied by a translocation of ACF towards the nucleus under normoxic conditions, which was further characterized by altered cell morphology that entailed cell shrinkage and blebbing (Figure 3B). Hypoxia (in the absence of PDT) triggered prominent translocation of ACF from the cytosol to the nucleus (Figure 3C). Interestingly, PDT-treated SK-ChA-1 cells that were placed in a hypoxic environment revealed a similar ACF distribution pattern as PDT-treated cells under normoxic conditions. ACF was mainly found in the nucleus in PDT-treated hypoxic cells, albeit at relatively lower levels compared to untreated hypoxic cells (Figure 3D).

### ACF potentiated PDT efficacy

**Figure 4:**
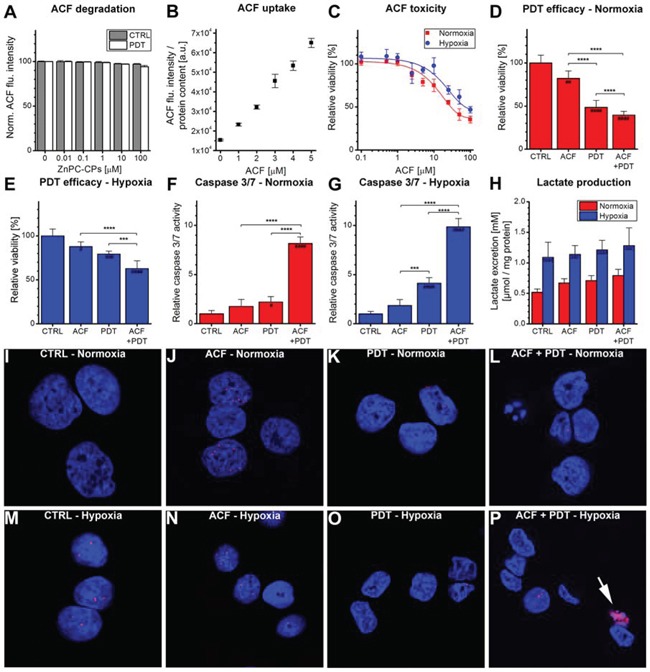
Combination treatment of ACF with PDT **A.** Evaluation of ACF stability using increasing concentrations of ZnPC-containing cell phantoms (ZnPC-CPs) with or without irradiation. ACF degradation was monitored using fluorescence spectroscopy (*n* = 4 per concentration). **B.** Cells were incubated with ACF for 24 hours, after which the uptake of ACF was determined using fluorescence spectroscopy. Data were normalized to protein content (*n* = 4 per concentration). **C.** ACF toxicity was determined after 24-hour incubation under either normoxic (red line) or hypoxic (blue line) conditions using the WST-1 method (*n* = 4 per group). Treatment efficacy of ACF and ACF + PDT was tested in SK-ChA-1 cells after 4 hours at **D.** normoxic and **E.** hypoxic culture conditions (*n* = 6 per group). (F, G) Relative caspase 3/7 activity was determined 4 hours after PDT at incubation at **F.** normoxic or **G.** hypoxic culture conditions (*n* = 6 per group). **H.** Lactate production by SK-ChA-1 cells treated with ACF and ACF + PDT was evaluated after 24 hours at normoxic (red bars) or hypoxic (blue bars) culture conditions (*n* = 6 per group). **I–P.** Analysis of DNA damage after control (CTRL), ACF, PDT, and ACF + PDT treatment. Cells were kept for 4 hours under normoxic (I-L) or hypoxic conditions (M-P) post-treatment. Cells were stained with DAPI (nuclei, blue) and phospho-H2AX (DNA double-strand breaks, red). The arrowhead in panel P indicates apoptosis. Readers are referred to the experimental section for the significance of the statistical symbols.

For clinical application purposes, ACF should remain stable during the application of PDT and during conditions of oxidative stress in order to inhibit HIF-1 activation after PDT and the subsequent microvascular shutdown. A model system was therefore used to study the stability of ACF during PDT. ACF was dissolved in buffer solution and exposed to increasing amounts of cell phantoms (*i.e*., artificial cells) loaded with ZnPC, of which it was demonstrated that ROS is produced upon irradiation [36]. As shown in Figure 4A, the application of PDT only marginally affected ACF fluorescence, confirming that ACF remained stable during illumination and conditions of hyperoxidative stress.

To determine the most suitable concentration of ACF for the improvement of PDT efficacy, the concentration-dependent uptake and toxicity of ACF were tested in SK-ChA-1 cells. ACF uptake followed a concentration-dependent linear pattern up to 5 μM ACF (Figure 4B). The toxicity of ACF was determined during a 24-hour incubation period under either normoxic or hypoxic conditions (Figure 4C). The IC_50_ concentration during normoxia and hypoxia, determined with the WST-1 assay, was 29 and 73 μM, respectively. Inasmuch as SK-ChA-1 cells exhibited a relative viability of ∼90% at 3 μM ACF during normoxia, this concentration was used in the rest of the experiments.

Next, SK-ChA-1 cells were incubated with ACF for 24 hours under normoxic conditions and treated with PDT (Figure 4D) to investigate ACF's adjuvant efficacy. As indicated, ACF was mildly toxic, which translated to slightly increased cytotoxicity when combined with PDT and normoxic incubation (Figure 4D). A similar trend was observed in cells that were maintained under hypoxic conditions after PDT (Figure 4E). In addition, the levels of caspase 3 and 7 (*i.e*., apoptosis markers) were assayed 4 hours post-treatment (Figure 4F, 4G). Under normoxic conditions, neither ACF nor PDT significantly affected caspase 3/7 levels, however, ACF + PDT resulted in a 8-fold higher caspase 3/7 activity in SK-ChA-1 cells (Figure 4G). During hypoxia, PDT resulted in a 4-fold increase in caspase 3/7 activity and ACF + PDT resulted in a 10-fold higher caspase 3/7 activity, indicating that apoptosis constitutes an important mode of cell death following combination treatment of ACF + PDT. None of the conditions induced the formation of DNA double-strand breaks, as assessed by a phospho-H2AX staining 4 hours after treatment (Figure 4I–4P), indicating that neither hypoxia nor ACF or PDT induce direct damage to DNA in the acute phase.

Lastly, inasmuch as HIF-1 signaling is a driving force behind glycolysis and the consequent production of lactate [49], the production of lactate was quantified in the cell culture medium 24 hours after ACF and PDT treatment (Figure 4H). Lactate excretion levels were substantially increased under hypoxic conditions in all treatment groups compared to normoxic cells, validating our hypoxic incubation model. However, no further intergroup differences were observed in this cell line with respect to lactate production. Neither ACF nor PDT therefore induced notable metabolic catastrophe in cells.

### ACF interferes with the regulation of HIF-1-induced target genes

**Figure 5:**
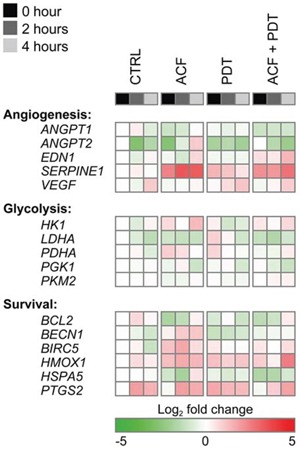
Gene expression analysis after control (CTRL), ACF, PDT, and ACF + PDT treatment Gene expression levels were obtained by qRT-PCR from SK-ChA-1 cells as analyzed 0 hours, 2 hours, or 4 hours post-treatment under hypoxic conditions. The plotted heat map data represents the log_2_-transformed fold change of each data point in relation to the 0-hour normoxic CTRL. Upregulated genes are depicted in red, downregulated genes in green. Numeric values are provided in Table S2.

To study whether pre-treatment with ACF influences post-PDT HIF-1α signaling, SK-ChA-1 cells were incubated with ACF and subsequently treated with PDT and maintained under hypoxic conditions. HIF-1 downstream targets were clustered in angiogenesis-, glycolysis-, and survival-associated genes and analyzed by quantitative reverse transcriptase polymerase chain reaction (qRT-PCR) at different time points after PDT (Figure 5). Moreover, additional HIF-1 target genes were included in the ACF-related transcriptomic analysis.

PDT induced the expression of *VEGF, HMOX1*, and *PTGS2*, corroborating the data in Figure 2C. ACF reduced the degree of *PTGS2* upregulation (only in the 0-h and 2-h group) and *VEGF* transcription post-PDT. Conversely, *EDN1* was downregulated by hypoxia and PDT but upregulated by ACF. In addition, *SERPINE1* was highly induced upon ACF treatment - an effect that was also observed after PDT in the presence of ACF. Altogether, these findings indicate that ACF by itself and in combination with PDT modulates several important HIF-1-induced transcriptional targets. However, the direction of the regulation is not always consistent within one functional class.

### Long-term exposure to ACF causes cell cycle arrest and apoptosis

**Figure 6:**
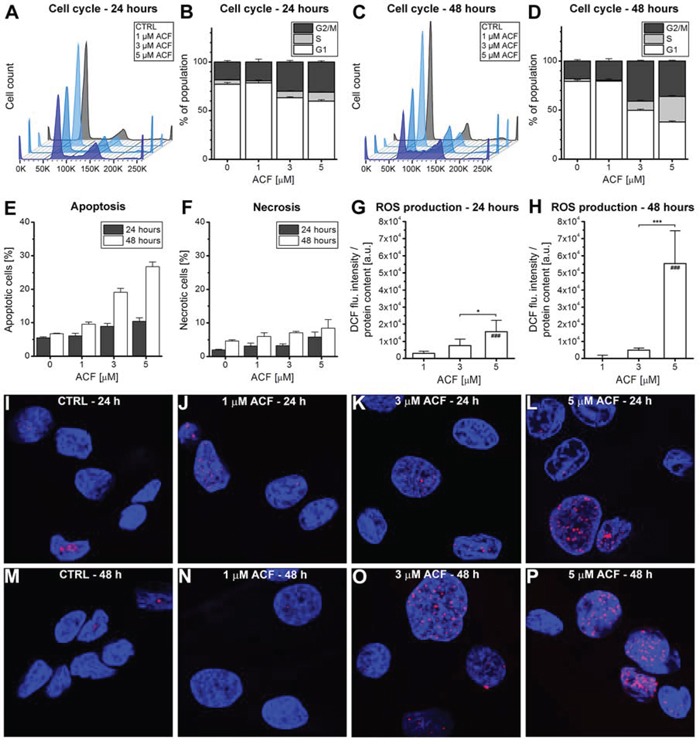
Response to ACF after prolonged exposure **A–D.** SK-ChA-1 cells were incubated with ACF for either (A, B) 24 hours or (C, D) 48 hours, after which the cell cycle profile was analyzed with flow cytrometry using propidium iodide staining (*n* = 3 per group). **E.** Flow cytometric analysis of SK-ChA-1 cells that were incubated with ACF for either 24 hours (in grey) or 48 hours (in white), after which the fraction of apoptotic (annexin V-positive) and **F.** necrotic (TO-PRO-3-positive) cells was determined (*n* = 3 per group). (G, H) SK-ChA-1 cells were exposed to ACF for **G.** 24 hours or **H.** 48 hours and intracellular DCF fluorescence was determined as a measure of ROS production. **I–P.** Analysis of DNA damage after control (CTRL) or ACF treatment. SK-ChA-1 cells received ACF or CTRL treatment for (I-L) 24 hours or (M-P) 48 hours, and were subsequently stained with DAPI (nuclei, blue) and phospho-H2AX (DNA double-strand breaks, red).

Although ACF is generally considered a specific HIF-1α/HIF-1β dimerization inhibitor [37], Hassan *et al*. have reported that ACF may also act as a dual topoisomerase I/II inhibitor [41]. Topoisomerase I/II inhibition is associated with cell cycle arrest and consequent apoptosis as a result of DNA double-strand breaks (reviewed in [42, 50]). In the acute phase after PDT, DNA double-strand breaks were not observed (Figure 4I–4P) but apoptotic signaling was pronounced, particularly in the ACF + PDT and hypoxia groups (Figure 4F and 4G). To investigate the potential topoisomerase I/II inhibitory effects, SK-ChA-1 cells were exposed to ACF for longer time frames (24 and 48 hours) under normoxic conditions, after which the cell cycle profile was analyzed using propidium iodide staining (Figure 6A–6D).

As shown in Figure 6B and 6D, ACF led to an increased fraction of cells in both the S- and G2/M-phase after 24 and 48 hours of incubation. The most significant effect of ACF was characterized by cell cycle arrest in the S-phase after 48 hours of incubation. Furthermore, ACF treatment was associated with increased apoptosis, but not necrosis, after 24 and 48 hours (Figure 6E, 6F), which concurred with elevated ROS production in cells (Figure 6G, 6H). Finally, incubation of SK-ChA-1 cells with ACF for 24 or 48 hours led to the formation of DNA double-strand breaks (Figure 6I–6P), although not in a concentration-dependent manner.

## DISCUSSION

Perihilar cholangiocarcinoma is a relatively rare cancer that is non-resectable in 70–80% of patients at the time of diagnosis [51]. Although PDT is not curative in these patients, the treatment does prolong the median survival of 6–9 months (stenting) to 21 months post-diagnosis (stenting + PDT) [9]. Driven by these promising results, novel avenues are being explored to enhance PDT efficacy in these refractory and rather lethal cancers. PDT is associated with microvasculature shutdown and consequent HIF-1 signaling that may contribute to therapeutic recalcitrance [10]. Therefore, this study was conducted to investigate the expression of HIF-1-induced proteins in perihilar cholangiocarcinomas to gauge whether inhibition of HIF-1 may be exploited as a therapeutic target in the context of PDT. Histological analysis revealed that human perihilar cholangiocarcinomas overexpress VEGF homogeneously and GLUT-1 heterogeneously and are replete with neoangiogenic vessels in the desmoplastic tissue, suggesting that HIF-1 is constitutively active in these tumors. Second, PDT of SK-ChA-1 cells with ZnPC-encapsulating liposomes caused HIF-1α stabilization and transcriptional upregulation of downstream targets of HIF-1. Third, ACF was taken up by SK-ChA-1 cells, especially during hypoxia, and translocated to the nucleus upon hypoxia and PDT. Lastly, ACF pretreatment was associated with S-phase cell cycle arrest and apoptosis and enhanced PDT efficacy, likely via inhibition of HIF-1 inhibition and topoisomerase I/II.

HIF-1α stabilization after PDT has been observed in various experimental settings. Ferrario *et al*. revealed that porfimer sodium-PDT resulted in HIF-1α stabilization in murine Kaposi's sarcoma [30]. PDT also upregulated the HIF-1-associated targets VEGF and COX-2 [30]. In mouse mammary carcinoma (BA) xenografts [31], porfimer-PDT led to an increase in HIF-1α, BIRC5, and VEGF protein levels. Lastly, murine mammary carcinoma (EMT-6) cells that were treated with porfimer sodium-PDT exhibited HIF-1α stabilization and its consequent translocation to the nucleus [28]. In line with these findings, our study demonstrated that HIF-1α was stabilized in SK-ChA-1 cells after incubation in a hypoxic chamber (to mimic vascular shutdown) and after PDT. PDT also led to the differential regulation of HIF-1-regulated genes, including *VEGF, PTGS2, SERPINE1, HMOX1*, and *BIRC5*. Consistent with these results, it was recently demonstrated that SK-ChA-1 cells subjected to sublethal PDT with neutral ZnPC-encapsulating liposomes significantly upregulated HIF-1-associated genes 90 minutes post-PDT [52]. Altogether, these findings attest that HIF-1α is activated following PDT and that this transcription factor constitutes an important therapeutic target, particularly in light of the fact that HIF-1 regulates biological processes that are important in PDT, such as glycolysis, angiogenesis, and survival [10].

The combinatorial use of HIF-1 inhibitors with PDT is a relatively new concept. For instance, Chen *et al*. used HIF-1α siRNAs in combination with Photosan-PDT in a head-and-neck cancer mouse model, which resulted in regression of tumor volume by ∼40% within 10 days [53]. Besides HIF-1 inhibition, its downstream target VEGF has been inhibited in various studies [32, 54, 55], which generally led to improved therapeutic efficacy. Although downstream targets of HIF-1 may be inhibited, from a pharmacology point of view it would be more attractive to inhibit HIF-1 itself, inasmuch as all the downstream targets are blocked concomitantly. As such, the HIF-1α/HIF-1β dimerization inhibitor ACF was evaluated for its adjuvant potential in SK-ChA-1 cells. ACF specifically binds the PER-ARNT-SIM (PAS) domain of HIF-1α and HIF-2α, which prevents the dimerization of HIF-1, thereby deterring its activation [37]. It was observed that ACF was taken up by SK-ChA-1 cells and translocated to the nucleus after hypoxia and/or PDT, presumably due to its binding to HIF-1α and HIF-2α. Moreover, ACF remained stable during the application of intense (laser) light exposure as well as during conditions of oxidative stress, suggesting that ACF will be able to inhibit HIF-1 after PDT and consequent vascular shutdown. The IC_50_ value of ACF in SK-ChA-1 cells was 29 μM during normoxia, which is in the range that has been observed for other cell lines [56]. Strese *et al*. found that human leukemic monocyte lymphoma (U937) was most susceptible to ACF, as demonstrated by an IC_50_ value of 4.6 μM, whereas human breast cancer (MCF-7) cells exhibited an IC_50_ value of 61 μM [56]. Pretreatment of SK-ChA-1 cells with ACF significantly improved therapeutic efficacy, which was partially mediated by the increase in caspase 3/7 levels (apoptosis).

In addition to HIF-1 inhibition, ACF has also been shown to act as a dual topoisomerase I/II inhibitor [41]. Of note, topoisomerase inhibitors (*e.g.*, topotecan [57]) may also repress gene transcription, but to what extent ACF is able to inhibit HIF-1-mediated signaling via this mechanism is currently elusive. Topoisomerase class I and II inhibitors cleave either one or both strands of DNA, respectively. Both topoisomerase I and II inhibitors may induce the formation of DNA double-strand breaks, inasmuch as the single-strand break that is induced by topoisomerase I inhibitors may turn into a double-strand break when the topoisomerase I cleavable complex collides with the replication fork [58]. This type of DNA damage may culminate in cell cycle arrest via tumor protein 53 (p53)-mediated p21^WAF1/CIP1^ induction, cellular senescence, and both p53-dependent and p53-independent apoptosis [42, 50, 58, 59]. To determine whether the observed cell death could (in part) be explained by topoisomerase I/II inhibition, SK-ChA-1 cells were incubated with ACF for 24 and 48 hours (*i.e*., a full cell cycle requires 48 hours [34]). It should be noted that, although SK-ChA-1 cells have a mutation (at codon 282) in the DNA binding domain of p53 [60], their p53 is still functional. ACF incubation led to cell cycle arrest in both the S-phase and the G2/M-phase and was associated with an increased percentage of apoptotic cells. SK-ChA-1 cells also exhibited DNA double-strand breaks as a result of ACF incubation. Collectively, these findings support the notion that ACF exhibits topoisomerase I/II inhibition activity that may contribute to greater therapeutic efficacy.

An interesting finding of this study is the upregulation of *SERPINE1* after ACF treatment. *SERPINE1* is a downstream target of both HIF-1 and p53 [61] and its protein product plasminogen activator inhibitor 1 (PAI1) is known to exhibit pleiotropic effects. PAI1 is involved in the inhibition of extracellular matrix remodeling, but it also has anti-apoptotic and pro-proliferative capacities and is involved in angiogenesis [62, 63]. This has been exemplified by Devy *et al*., who demonstrated that cultured mouse aortic rings from PAI1-deficient mice, which were stimulated with PAI1, exhibited a dose-dependent angiogenic response [64]. Whereas low-dose levels of PAI1 were associated with increased angiogenesis, high-dose levels of PAI1 inhibited microvessel formation [64]. To what extent p53 is responsible for *SERPINE1* induction after ACF treatment is currently elusive, as are the consequences of PAI1 induction in the context of PDT.

As stated earlier, the use of inhibitors of specific survival pathways with PDT is a relatively novel strategy. Several studies have indicated that inhibition of survival pathways in conjunction with PDT may be an attractive means to enhance PDT efficacy (reviewed in [10]). Consistent with these results, the present findings also encourage the use of small molecule inhibitors (*e.g.*, HIF-1 inhibitors) of survival pathways together with PDT. These small molecule inhibitors can be co-encapsulated with a photosensitizer into a single drug delivery system, such as liposomes, in order to improve treatment outcome.

In conclusion, HIF-1 is overexpressed in a variety of solid cancers and is often associated with therapeutic recalcitrance, inasmuch as it stimulates glycolysis, angiogenesis, and survival. This study demonstrated that HIF-1 inhibition via ACF may be an attractive method to potentiate PDT efficacy in perihilar cholangiocarcinoma. Interestingly, not only HIF-1 inhibition, but also topoisomerase I/II inhibition by ACF may further contribute to increased PDT efficacy. *In vivo* studies as addressed in [65] are necessary to validate the potential of ACF in combination with PDT.

## MATERIALS AND METHODS

### Chemicals

1,2-dipalmitoyl-*sn*-glycero-3-phosphocholine (DPPC), 1,2-dipalmitoyl-*sn*-glycero-3-phospho-L-serine (DPPS), and 3β-[N-(N’,N’-dimethylaminoethane)-carbimoyl]cholesterol (DC-chol) were purchased from Avanti Polar Lipids (Alabaster, AL). L-α-phosphatidylethanolamine, distearoyl methoxypolyethylene glycol conjugate (DSPE-PEG, average PEG molecular mass of 2,000 amu), ZnPC, 4-(2-hydroxyethyl)-1-piperazineethanesulfonic acid (HEPES), fibronectin, sodium chloride (NaCl), β-mercaptoethanol, cholesterol, chloroform, Nile Red, paraformaldehyde, sucrose, bovine serum albumin (BSA), Tween 20, CoCl_2_, ACF, and pyridine were obtained from Sigma-Aldrich (St. Louis, MO). Tris-HCl and dimethyl sulfoxide (DMSO) were purchased from Merck KgaA (Darmstadt, Germany). Ethanol was obtained from Biosolve (Valkenswaard, the Netherlands). Protease inhibitor cocktail and water-soluble tetrazolium-1 (WST-1) were purchased from Roche Applied Science (Basel, Switzerland). 2′,7′-dichlorodihydrofluorescein diacetate (DCFH_2_-DA) was obtained from Life Technologies (Carlsbad, CA).

All lipids were dissolved in chloroform, purged with nitrogen gas, and stored at −20°C. Phospholipid stock concentrations were determined by the inorganic phosphate assay modified from [66]. ZnPC was dissolved in pyridine at a 178-μM concentration and stored at room temperature (RT) in the dark, CoCl_2_ was dissolved in MilliQ at a concentration of 50 mM, and ACF and DCFH_2_-DA were dissolved in DMSO at a concentration of 50 mM.

### Histology

Histology was performed on two patient-derived, paraffin-embedded perihilar cholangiocarcinoma biopsies. Tissue sections were dewaxed in xylene and rehydrated in graded steps of ethanol. Endogenous peroxidase activity was blocked with methanol containing 0.3% peroxide (20 min, RT). Heat-induced epitope retrieval (HIER) was performed in a pretreatment module (Thermo Fisher Scientific, Fremont, CA) using Tris-EDTA (VEGF, Ki-67, CD31, GLUT-1) or citrate buffer (CD105) for 20 minutes at 98°C. Throughout the staining procedure all washing steps were performed with Tris-buffered saline. Superblock (Immunologic, Duiven, the Netherlands) was applied as a protein block prior to staining with primary antibodies.

All antibodies were diluted with antibody diluent (Scytek, Logan, UT). Single stains for CD105 (rabbit anti-human, polyclonal, cat # RB-9291, Thermo Fischer Scientific), VEGF (rabbit anti-human, polyclonal, cat. # sc-152, Santa Cruz Biotechnology, Santa Cruz, CA), and GLUT-1 (rabbit anti-human, polyclonal, cat. # RB-9052, Thermo Fischer Scientific) were performed. These primary antibodies were visualized with BrightVision HRP-conjugated anti-rabbit polymer (Immunologic) and BrightDAB. The sections were counterstained with hematoxylin.

Sequential double staining [67] was performed for CD31 (mouse anti-human, clone JC70A, cat. # M0823, Dako, Glostrup, Denmark) and Ki-67 (rabbit anti-human, clone SP6, cat. # RM9106, Thermo Fischer Scientific). Ki-67 was visualized with BrightVision AP-conjugated anti-rabbit polymer (Immunologic) and Vector Red (Vector Labs, Burlingame, CA). Next, an intermediate HIER step using Tris-EDTA buffer (10 minutes at 98°C) was applied to remove all antibodies but leaving the chromogen intact [68]. Finally, CD31 was visualized with BrightVision AP-conjugated anti-mouse polymer (Immunologic) and PermaBlue plus/AP (Diagnostics Biosystems, Pleasanton, CA). All slides were dried on a hotplate (50°C) and permanently mounted with Vectamount (Vector Labs).

It should be noted that, as part of the clinical diagnostics protocol at the Department of Pathology, all antibodies had been validated for their cross-reactivity and immunohistological staining efficacy using tissue that overexpresses the respective marker. Immunostaining for HIF-1α directly was not performed due to the instability of the HIF-1α antigen (degrades within a few minutes after biopsy).

### Liposome preparation

ZnPC-ETLs were composed of DPPC:DC-chol:cholesterol:DSPE-PEG (66:25:5:4, molar ratio) and ZnPC was incorporated at a ZnPC:lipid molar ratio of 0.003. Liposomes were prepared using the lipid film hydration technique as described previously [36]. ZnPC-ETLs were characterized for size and polydispersity by photon correlation spectroscopy (Zetasizer 3000, Malvern Instruments, Malvern, Worcestershire, UK). Liposomes were purged with nitrogen and stored in the dark at 4°C until use.

### Cell culture

Human perihilar cholangiocarcinoma (SK-ChA-1) cells were grown at standard culture conditions (37°C, 5% CO_2_, and 95% air) and cultured in Roswell Park Memorial Institute (RPMI) 1640 culture medium supplemented with 10% fetal bovine serum (FBS) (v/v) (both from Gibco, Invitrogen, Carlsbad, CA), 1% penicillin/streptomycin (v/v), 1% L-glutamine (v/v) (both from Lonza, Walkersville, MD), and 1 × 10^−5^% β-mercaptoethanol (v/v) (Sigma-Aldrich). Cells were passaged weekly at a 1:10 ratio. For all experiments, SK-ChA-1 cells were seeded in 24-wells (500 μL/well) or 6-wells plates (2 mL/well) (Corning, Corning, NY) at a density of 2 × 10^5^ cells/mL. Confluent monolayers were achieved 48 hours after cell seeding, whereas 70–80% confluency was reached 24 hours after cell seeding.

### PDT protocol

Cells were seeded in either 24-wells or 6-wells plates as indicated in the specific subsections and cultured until confluence. In case of ACF pre-treatment, cells were incubated with 3 μM ACF (in serum-free supplemented RPMI 1640 medium) for 24 hours prior to PDT. Next, cells were washed with PBS and incubated with ZnPC-ETLs (in serum-free supplemented RPMI 1640 medium) for 1 hour at standard culture conditions. Cells were washed twice with PBS and fresh serum- and phenol red-free supplemented RPMI 1640 was added to the cells. Serum was deliberately withdrawn after PDT in order to emulate the hyponutritional status of PDT-treated tumor cells *in vivo*, which is caused by the vascular shutdown. PDT was performed with a 671-nm solid state diode laser (CNI Laser, Changchun, China) at a power of 500 mW to achieve a cumulative radiant exposure of 15 J/cm^2^. After PDT, cells were either placed at standard culture conditions (normoxia) or placed in a hypoxic chamber [69] (hypoxia) to mimic vascular shutdown.

### Cell viability

Cell viability was assessed using the WST-1 assay as described previously [36].

### Western blotting

Cells were seeded in 6-wells plates and cultured until confluence. Cells were incubated with 10 μM ZnPC-ETLs (final lipid concentration) and treated with PDT (section “*PDT protocol*”). At 0, 30, 60, 120, and 240 minutes after PDT, cells were placed on ice and lysed in ice-cold Laemmli buffer [70] supplemented with protease inhibitor cocktail (1 tablet per 5 mL buffer). A 20-hour incubation with 500 μM CoCl_2_ served as a positive control for HIF-1α stabilization [71]. The lysates were passed 10 × through a 25-gauge needle (BD Biosciences, San Jose, CA) to shear DNA. Next, samples were placed in a heat block for 10 minutes at 95°C, after which the samples were centrifuged for 15 minutes at 13,000 × g (4°C). Samples (30 μg) were loaded on a 10% SDS-PAGE precast gel (50 μL slot volume, Bio-Rad Laboratories, Hercules, CA) and the electrophoresis was performed for 90 minutes at 125 V. The gels were blotted onto methanol-primed PVDF membranes (Millipore, Billerica, MA) for 1 hour at 330 V at 4°C. Protein membranes were blocked for 1 hour with 5% dried milk powder (Protifar, Nutricia, Cuijk, the Netherlands) in 0.2% Tween 20 Tris-buffered saline (TBST, 20 mM Tris-HCl, 150 mM NaCl, pH = 7.5). The membranes were incubated overnight at 4°C on a rocker with anti-HIF-1α (1:500, clone 54/HIF-1α, BD Transduction Laboratories (Franklin Lakes, NJ)) and anti-β-actin (1:4,000, AC-74, Sigma-Aldrich). Next, the membranes were washed 4 times in TBST and incubated with HRP-conjugated goat-anti-mouse IgG_1_ (1:1,000, Dako Cytomation (Glostrup, Denmark)) for 1 hour at RT. Subsequently, membranes were washed 3 times with TBST and 2 times with TBS. The enhanced chemiluminescence (ECL) kit (Thermo Scientific) was used as substrate for β-actin and ECL plus (Thermo Scientific) was used as substrate for HIF-1α. Protein bands were visualized on an ImageQuant LAS 3000 luminometer (GE Healthcare, Little Chalfont, UK).

### qRT-PCR

Cells were seeded in 6-wells plates and treated with PDT as described in the section “*PDT protocol*”. RNA was extracted using TRIzol according to the manufacturer's protocol (Life Technologies). RNA was quantified and analyzed with a Nanodrop 2000 UV-VIS spectrophotometer (Thermo Scientific). cDNA synthesis and RT-qPCR reactions were performed according to [52]. The primers that were used in this study are listed in Table S1. The data was analyzed using the LinRegPCR software in which relative starting concentrations of each cDNA template (*N*_0_) were calculated [72], after which the *N*_0_ values of the target genes were corrected for the respective *N*_0_ of the S18 rRNA. All S18 rRNA-corrected *N*_0_ values of each gene were compared to the average *N*_0_ of the untreated normoxic control samples. A log_2_ transformation was performed in order to obtain absolute fold-differences in expression levels of the genes of interest.

### Confocal microscopy

Microscope cover slips (24 × 40 mm, VWR, Lutterworth, UK) were first coated with 5 × 10^−4^% (w/v) fibronectin in 0.9% NaCl (Fresenius Kabi, Bad Homburg, Germany) for 2 hours at 37°C. Next, the fibronectin-containing solution was aspirated and cells were seeded and allowed to grow overnight. To determine the ACF subcellular localization, cells were either untreated or subjected to PDT as described in the section “*PDT protocol*”, and subsequently incubated with 3 μM ACF for 4 hours under normoxia and hypoxia as specified. Next, cells were washed with 1 mL of PBS and fixed with a mixture of 4% paraformaldehyde and 0.2% sucrose for 5 min. Cells were washed with 1 mL of PBS and stained with 1 μM Nile Red (in PBS) for 1 min. Cells were washed thrice with 1 mL PBS and mounted on microscope slides using Vectashield mounting medium (Vector Laboratories, Burlingame, CA). After 1 h, the slides were sealed with nail polish.

For the assessment of DNA damage, cells were fixed with a mixture of 4% paraformaldehyde and 0.2% sucrose for 5 min and permeabilized in 0.1% TX-100 (in PBS) for 5 min. Next, cells were washed with 1 mL of PBS and incubated for 16 hours with mouse anti-human phospho-H2AX-AlexaFluor647 (Cell Signaling Technology, Danvers, MA) at a 1:100 dilution in 0.5% BSA and 0.15% glycine (in PBS, staining buffer) at 4°C. Next, cells were washed thrice with staining buffer and mounted on microscope slides using Vectashield mounting medium with 4′,6-diamidino-2-phenylindole (DAPI) (Vector Laboratories). After 1 h, the slides were sealed with nail polish.

Cells were imaged on a Leica SP8 laser scanning confocal microscopy system (Leica Microsystems, Wetzlar, Germany). Fluorescence intensities were measured at λ_ex_ = 405 nm, λ_em_ = 415–480 nm for DAPI, λ_ex_ = 470 nm, λ_em_ = 480–550 nm for ACF, λ_ex_ = 540 nm, λ_em_ = 550–650 nm for Nile Red, and λ_ex_ = 660 nm, λ_em_ = 670–750 nm for phospho-H2AX. All experiments were performed using the same laser and microscope hardware settings.

### ACF degradation

To evaluate the stability of ACF during PDT, 450 μL of ACF (80 μM) in serum-free and phenol red-free RPMI 1640 medium was added to 24-wells plates. Next, 50 μL of increasing concentrations of ZnPC-containing cell phantoms (85% DPPC, 10% DPPS, 5% cholesterol, molar ratio; ZnPC:lipid ratio of 0.003) in physiological buffer (10 mM HEPES, 0.88% (w/v) NaCl, pH = 7.4, 0.292 osmol/kg) was added to the wells. The baseline ACF fluorescence was read at λ_ex_ = 460 ± 40 nm and λ_em_ = 520 ± 520 nm using a BioTek Synergy HT multi-well plate reader (Winooski, VT). Subsequently, the cells were subjected to PDT (500 mW, 15 J/cm^2^) and ACF fluorescence was determined as a measure of ACF degradation. The data was normalized to control wells (*n* = 4 per group).

### ACF uptake

Cells were cultured in 24-wells plates until confluence. Cells were washed with PBS and incubated with ACF in supplemented serum-free RPMI 1640 medium for 24 hours. After incubation, cells were washed with PBS and fresh supplemented serum-free RPMI 1640 medium was added to the wells. Next, ACF fluorescence, as a measure of uptake, was read at λ_ex_ = 460 ± 40 nm and λ_em_ = 520 ± 520 nm using a BioTek Synergy HT multi-well plate reader. Data were normalized to protein content per well (*n* = 4 per group) as determined with the SRB assay [73].

### Caspase 3/7 activity

Cells were cultured in 24-wells plates and subjected to treatment as described above. Cells were incubated in 200 μL of serum- and phenol-red free medium and maintained at either normoxic or hypoxic conditions for 3.5 hours post-treatment. After treatment and normoxic/hypoxic incubation, 25 μL of Caspase-Glo assay reagent (Promega, Madison, WI) was added and cells were incubated for 30 minutes under the aforementioned conditions. Luminescence was read on a BioTek Synergy HT multiplate reader at 560 ± 20 nm and a signal integration time of 1 s. Data were obtained from n = 5 measurements and corrected for background luminescence.

### Lactate production

Cells were cultured in 24-wells plates until confluence and treated with ACF and PDT as indicated in the section “PDT protocol”. After 24 hours, extracellular lactate levels were determined using The Edge blood lactate analyzer (Apex Biotechnology, Hsinchu, Taiwan). Lactate concentrations were determined from a standard curve and corrected for the average protein content per group as determined with the bicinchoninic acid assay (Thermo Scientific).

### Flow cytometry

For cell cycle analysis, cells were seeded in 6-wells plates and cultured until 70–80% confluence. Cells were incubated with ACF (in supplemented serum-free RPMI 1640 medium) for 24 or 48 hours, after which cell cycle analysis was performed using flow cytometry according to ref. 69.

The mode of cell death following ACF incubation was analyzed by flow cytometry using APC-conjugated Annexin V (eBioscience, San Diego, CA) for apoptosis and TO-PRO-3 (Life Technologies) for necrosis. Cells were seeded in 6-wells plates and cultured until 70–80% confluence. Next, cells were incubated with ACF (in supplemented serum-free RPMI 1640 medium) for 24 or 48 hours. After incubation, the samples were prepared as described previously [36] and assayed on a FACSCanto II (Becton Dickinson, Franklin Lakes, NJ). Ten thousand events were recorded in the gated region and data was analyzed using FlowJo software (Treestar, Ashland, OR).

### Intracellular ROS assay

Cells were seeded in 24-wells plates and cultured until 70–80% confluence. Thereafter, cells were washed with PBS and incubated with ACF or vehicle (DMSO) in supplemented serum-free RPMI 1640 medium for 24 or 48 hours. After the indicated time points, the medium was removed, cells were washed with serum- and phenol red-free RPMI 1640 medium, and cells were incubated with 100 μM DCFH_2_-DA (in serum- and phenol red-free RPMI 1640 medium) for 1 hour at standard culture conditions. Next, cells were washed with serum- and phenol red-free RPMI 1640 medium, and fresh serum- and phenol red-free RPMI 1640 medium was added to the wells. Intracellular 2′,7′-dichlorofluorescein (DCF) fluorescence, which is a measure of ROS production, was read on a BioTek Synergy HT multiplate reader at λ_ex_ = 460 ± 40 nm and λ_em_ = 520 ± 520 nm. Data were obtained from n = 6 measurements and corrected for ACF fluorescence, protein content using the SRB assay, and DCF fluorescence (basal metabolic rate) of control cells.

### Statistical analysis

Data were analyzed in GraphPad Prism software (GraphPad Software, San Diego, CA). Data were analyzed for normality using a Kolmogorov-Smirnov test. Normally distributed data sets were analyzed with either a student's *t*-test or a one-way ANOVA and subsequent Bonferroni post-hoc test. Non-Gaussian data were statistically analyzed using a Mann-Whitney U or Kruskal-Wallis test and a Dunn's post-hoc test. All data are reported as mean ± standard deviation. In the figures, intergroup differences are indicated with (*) and differences between treated groups versus the untreated (CTRL) group are indicated with (#). Differences between normoxic and hypoxic data are, when relevant, indicated with ($). The level of significance is reflected by a single (*p* < 0.05), double (*p* < 0.01), triple (*p* < 0.005), or quadruple sign (*p* < 0.001).

## SUPPLEMENTARY FIGURES AND TABLES







## Abbreviations

ACF: acriflavine
ANGPT: angiopoietin
BIRC5: baculoviral inhibitor of apoptosis repeatcontaining 5
COX-2: cyclooxygenase 2
DAPI: 4′,6-diamidino-2-phenylindole
DC-chol: 3β-[N- (N′,N′-dimethylaminoethane)-carbimoyl]cholesterol
DCF: 2′,7′-dichlorofluorescein
DCFH2-DA: 2′,7′-dichlorodihydrofluorescein diacetate
DMSO: dimethyl sulfoxide
DPPC: 1,2-dipalmitoyl-snglycero- 3-phosphocholine
DPPS: 1,2-dipalmitoylsn- glycero-3-phospho-L-serine
DSPE-PEG: L-α- phosphatidylethanolamine, distearoyl methoxypolyethylene glycol conjugate
ECL: enhanced chemiluminescence
EDN1: endothelin-1
ER: endoplasmic reticulum
ETL: endothelium-targeted liposome
FIH: factor inhibiting HIF
GLUT-1: glucose transporter 1
HEPES: 4-(2-hydroxyethyl)-1-piperazineethanesulfonic acid
HIER: heat-induced epitope retrieval
HIF: hypoxia-inducible factor
HK1: hexokinase 1
HMOX1: heme oxygenase 1 (gene)
HO-1: heme oxygenase 1 (protein)
LDHA: lactate dehydrogenase A
NF-κB: nuclear factor κB
PAI1: plasminogen activator inhibitor 1
PAS: PER-ARNT-SIM
PHD: prolyl hydroxylase
PTGS2: prostaglandin synthase 2
qRT-PCR: quantitative reverse transcriptase polymerase chain reaction
ROS: reactive oxygen species
RT: room temperature
SERPINE1: serpin peptidase inhibitor, clade E
TBS: tris-buffered saline
TBST: TBS supplemented with 0.2% Tween-20
VEGF: vascular endothelial growth factor
VHL: Von Hippel-Lindau tumor suppressor protein
WST- 1: water-soluble tetrazolium-1
ZnPC: zinc phthalocyanine.
