# Supplementary material for: Inhibition of hypoxia inducible factor 1 and topoisomerase with acriflavine sensitizes perihilar cholangiocarcinomas to photodynamic therapy
Source: Oncotarget. 2015 Nov 27;7(3):3341–56. doi: 10.18632/oncotarget.6490 (PMC4823110; doi:10.18632/oncotarget.6490)
Supplement: Supplementary file 1 [file oncotarget-07-3341-s001.pdf]

## SUPPLEMENTARY TABLES

**Supplementary Table S1: Primer information for primer pairs used for qRT-PCR, including amplicon size, PCR efficiency, melting curve analysis, and electrophoretic analysis of the amplicon.** PCR efficiencies were categorized as low (<1.7), medium (1.7 - 1.8), or high (>1.8). Melting curves were analyzed for the formation of a single product (sp). Electrophoretic analysis was performed to assess the formation of single bands (sb), where the occurrence of a minor byproduct (mbp) was deemed acceptable.

**Supplementary Table S2: Overview of the mean, log2-transformed fold-changes in mRNA levels and standard deviations of selected target genes obtained in SK-ChA-1 cells.**
